# YPEL1 Inhibits Development of Gemcitabine Resistance in NK / T Cell Lymphomas

**DOI:** 10.2174/0115680096328745250122231110

**Published:** 2025-03-17

**Authors:** Miao Wang, Siyu Qian, Yue Zhang, Qingjiang Chen, Xudong Zhang, Mingzhi Zhang

**Affiliations:** 1 Department of Oncology, the First Affiliated Hospital, Zhengzhou University, Zhengzhou, Henan Province, China

**Keywords:** YPEL1, YT, cancer, tumor, YT/Gem-R, apoptosis

## Abstract

**Introduction:**

Yippee Like 1 (YPEL1) is a nuclear protein involved in various cellular processes, including cell cycle regulation, senescence, and mammalian development. It plays a dual role in cancer, functioning as either an antitumor or tumor-promoting factor.

**Methods:**

We assessed the RNA and protein levels of YPEL1 in both NK/T-cell，lymphoma cell lines and gemcitabine-resistant lymphoma cells using qPCR and western blot, respectively. Flow cytometry was employed to analyze cellular apoptosis and the cell cycle. The impact of YPEL1 knockdown on the proliferation of NK/T-cell lymphoma cells was evaluated through CCK8 assay and a xenograft model, while transcriptomic analysis was conducted to explore the cellular signaling pathways influenced by YPEL1 silencing.

**Results:**

In the current study, *via* The Cancer Genome Atlas (TCGA) search, we found that YPEL1 is aberrantly expressed in various cancers. High expression of YPEL1 correlated with poorer survival outcomes, whereas low expression of YPEL1 was associated with improved overall survival of patients. YT cell lines and gemcitabine-resistant YT cell line (YT/Gem-R) exhibit elevated levels of the YPEL1 protein.

**Conclusion:**

These findings suggest that YPEL1 exhibits the potential for being used as a target for NK / T cell lymphoma treatment

## 1 INTRODUCTION

The YPEL gene family, consisting of five members (YPEL1-YPEL5) is highly conserved across eukaryotic species [1]. These proteins are located near the mitotic apparatus [2, 3], indicating they may play a role in cell division. YPEL genes are involved in various biological processes, including cell cycle regulation, senescence, mammalian development, and tumor progression [4-8].

YPEL1 is a member of the YPEL proteins family [9] and is located on chromosome 22q11.2 [1]. It is a ubiquitous intracellular protein featuring a zinc finger-like metal-binding domain [10]. It regulates the behavior and cellular morphology of epithelial cells [9] and has been shown to induce apoptosis, contributing to dysmorphogenesis in the early avian craniofacial mesenchyme [11]. *In vitro*, overexpression assays in NIH3T3 cells indicate a potential role for YPEL1 in regulating cytoskeletal and adhesion machinery [12].

Notably, YPELs can function as either tumor promoters or inhibitors depending on cancer type. In pancreatic cancer cell lines, YPEL1 was found to be downregulated in malignant phenotype and during epithelial-mesenchymal transition [13]. Based on recent bioinformatic studies, YPEL1 showed high expression in non-small cell lung cancer samples treated with erlotinib, particularly in cases with epidermal growth factor receptor mutantions [12]. Furthermore, YEPL1 interacts with the LIM domain of LMO7, exacerbating spindle assembly checkpoint errors caused by LIM overexpression [14]. In pancreatic ductal adenocarcinoma cell lines and tissues, YEPL1 mRNA expression is significantly lower than its normal counterparts [15].

Similarly, YPEL1 has been shown to inhibit cell growth and invasiveness in gastric cancer and plays antitumor roles in glioma [2]. Conversely, the downregulation of YPEL1 in human colon cancer tissue reduced invasiveness and proliferation [16, 17], showcasing its oncogenic role. Notably, increased YPEL1 expression levels is associated with improved overall survival and disease-specific survival [18]. Thus, the function of YPEL1 could vary across different tumor types, acting as either an antitumor or oncogenic factor.

Given the controversial roles of YPEL1 in different cancers, understanding its precise role and effects on tumor biology is vital. In the current study, we analyzed YPEL1 deregulation across various cancers with data from The Cancer Genome Atlas (TCGA). Our findings indicate that lower YPEL1 expression is associated with better overall survival, whereas higher expression showed a correlation with poor clinical outcomes in patients. Furthermore, our results demonstrated that YPEL1 depletion significantly suppressed NK/T tumor growth *in vivo*.

## 2 MATERIALS AND METHODS

### 2.1 Cell Culture

The human NK cell leukemia cell YT cell lines were purchased from the cell bank of the Biological Company of China, Beijing. YT cells were maintained in RPMI-1640 medium supplemented with 10% fetal bovine serum (FBS). gemcitabine-resistant cell line (YT/Gem-R) cells were cultured in the same medium with an additional 0.2 mg/mL of gemcitabine. Both cell lines were cultured at 37℃ in a humidified incubator under standard controlled conditions with 5% CO_2_.

### 2.2 Establishment of YT/Gem-R

### 2.3 RNA Isolation and qPCR

Total RNA was extracted from control and treated cell lines using TRIzol and chloroform according to the manufacturer’s instructions. Briefly, cells were collected, rinsed with PBS, and then lysed using 1 ml of TRIzol reagent. For each 1 mL of TRIzol, 0.4 ml of chloroform was added, and mixed vigorously. The mixtures were then incubated at room temperature for 5 minutes and centrifuged at 12,000 rpm for 15 minutes at 4℃. Following centrifugation, the upper aqueous phase containing RNA was transferred to a new tube. Next, 0.5 mL of isopropanol was added and the sample was incubated for 10 minutes at room temperature before being centrifuged at 12000 rpm for 10 minutes at 4℃. The supernatants were discarded, and the RNA pellets were washed twice with 75% ethanol by centrifuging for 5 minutes at 4℃ at 7500 rpm. Lastly, the RNAse- and DNAse-free water was added to dissolve the RNA. A Nanodrop was used to determine the of quantity and quality of RNA samples.

We synthesized the cDNA from total RNA using random hexamers and oligo (dT) primers, according to the iScriptTM cDNA synthesis kit (Bio-rad, #1708891) protocol. The mRNA expression levels of candidate genes were quantified with the specific AceQ^®^ qPCR SYBR^®^ Green Master Mix on an ABI Prism 7500 system (Applied Biosystems). The qPCR cycling conditions were set to 95℃ for 10 min, followed by 40 cycles of 95 ℃ for 15 seconds and 60℃ for 60 seconds. All RT-qPCR experiments were conducted in triplicate for statistical reliability. The relative expression of the target genes was calculated using the 2^-ΔCt, with β-actin as the reference control. Primers used in this study are listed in Supplementary Table **S1**.

### 2.4 RNA Sequencing

Following Illumina's best practices, the RNA-Sequence (RNA-seq) experiments were performed by Shenzhen Huada Gene Technology Service Co., Ltd. Total RNA was isolated from YT cells and YT/Gem-R cells using the standard TRIzol reagent according to the manufacturer's instructions. Genomic DNA was removed with DNase I. mRNA was enriched with magnetic beads and oligo (dT), and a double-stranded cDNA libraries were constructed. To select the appropriate fragment size for PCR amplification, the ends of the cDNA were repaired and poly-A tails were added. mRNA-seq library was then sequenced on the Illumina HiSeq 2000 platform, and the data were subsequently analyzed by the Beijing Genomics Institute (BGI) platform.

### 2.5 Western Blot

Cells were lysed using RIPA buffer (Abcam#ab156034). Protein concentrations were determined using the BCA (Bicinchoninic Acid) assay. The lysates were mixed with 2× Protein loading buffer (Biorad#1610737EDU) and boiled at 100℃ for 10 minutes, subsequently, 20 μg of protein was loaded onto a 12% SDS PAGE gel. After the gel run, the proteins were transferred to a polyvinylidene difluoride (PVDF) membrane in a wet transfer method. Membranes were blocked for an hour at room temperature with 5% skimmed milk. then washed with TBST for 15 minutes and incubated the appropriate primary antibodies overnight at 4℃. Finally, the membranes were incubated in the HRP-conjugated secondary antibodies for 1 hour at room temperature. Immunoblots were visualized using a chemiluminescent substrate (Thermoscientific#34075), and the images were captured using the Thermo Scientific^TM^ equipment.

### 2.6 YPEL1 shRNA Interference

Lentiviruses expressing shRNAs targeting YPEL1 were targeted using HEK293FT cells. These cells were cotransfected with 2µg pLKO.1 shRNA control or pLKO.1-YPEL1, along with 2 µg of pGag/Pol/PRE, 1 µgpVSVG, and 2μg pREV. Fresh medium was added after transfection for 12 hours. 72 hours after transfection, the supernatants containing lentiviral particles were harvested. YT cells were then cultured with lentiviral particles along with 8μg/ml of polybrene (Sigma) for 24 hours. 48 hours after infection, 1µg/ml puromycin was used to establish stable cell lines. The knockdown efficiency was evaluated using real-time RT-PCR and Western blotting.

### 2.7 Cell Proliferation Assay

Approximately 5,000 YT cells with modifications (YT-shYPEL1) were seeded in 96-well plates with gemcitabine-containing medium and cultivated for different time points such as 24 hours, 48 hours, and 72 hours. Subsequently, we used the CCK-8 reagent to detect cell proliferation according to the manufacturer's instructions.

### 2.8 Immunohistochemistry

To determine the expression of YPEL1 in tissues, we performed immunohistochemical staining on 5-mm slices of paraffin-embedded Natural Killer/T-cell Lymphoma (NKTCL) tissues. The slides underwent a 25-minute heating at 60℃, followed by deparaffinization with xylene, rehydration through a graded alcohol series (at 100%, 95%, and 70%), and then placed in deionized water. Antigen retrieval was achieved by heating the slides in a 10 mM sodium citrate buffer (pH 6.0). Endogenous peroxidase activity was blocked with 3% H_2_O_2_ for 10 minutes. Subsequently, the slides were blocked in PBS containing 3% goat serum in PBS for 30 minutes, then incubated with the IHC-specific antibody YPEL1 at 4℃ overnight. After washing, the slides were treated with a polymer tagged with horseradish peroxidase (HRP) and goat anti-mouse immunoglobulins for 1 hour. The desired brown signal was developed using 3,3′-diaminobenzidine chromogen solutions and conducting the chromogenic reaction for 10 minutes. Slides were then washed, dehydrated, counterstained with hematoxylin, mounted with Vectamount mounting medium (Thermofisher Scientific), and covered. Images were captured using an Olympus LS microscope.

### 2.9 Measurement of Apoptosis

Cells were seeded at 1.5 × 10^5^ cells/ml in 6-well plates, and YT/Gam-R cells were cultured in a medium containing 0.2 μg/L gemcitabine and 2 μg/ml puromycin, while YT cells were cultured without gemcitabine. After 24 hours, cells were harvested and washed with cold PBS. Cells were suspended in 500 μL of binding buffer and stained with 5 μL of Annexin V-FITC and 5 μL of propidium iodide (PI) staining solution. The cell mixtures were gently mixed by pipetting and analyzed using the BD Accuri C6 flow cytometer to detect PI and Annexin V-FITC using the FL1 and FL3 channels, respectively. The data were analyzed using the BD Accuri C6 software.

### 2.10 *In Vivo* Mouse Model of Xenograft

Male BALB/c nude mice, aged 3-4 weeks, were purchased from Vitalstar Biotechnology (Beijing, China). Experimental and sampling procedures were approved by the ethical committee of the First Affiliated Hospital of Zhengzhou University (2021-KY-1025-002). The animal experiments were conducted in compliance with international guidelines and local regulations, the Guide for the Care and Use of Laboratory Animals，FELASA Guidelines， and 3Rs principle.

For the experiment, nude mice (n = 7 mice per group) received a subcutaneous injection of 2 × 10^6^ YT cells, either control or stably expressing shRNA for YPEL1 knockdown. At predetermined time points, mice were sacrificed, and tumors were excised from the injection sites. The excised tumors were immediately subjected to downstream analyses, including measurement of tumor volume, weight, and histological examination. Representative photographs of the tumors were taken to document their appearance and size. The results of these analyses were used to assess the impact of YPEL1 knockdown on tumor growth and progression.

### 2.11 Statistical Analysis

Statistical analyses of all experimental data were performed using the SPSS25.0 software. Data visualization was carried out using GraphPad 9.0 and Adobe Photoshop CS6. For each data set, the mean and standard deviation were calculated. Statistical significance was determined using either the t-test or the corrected t-test, P value *P* < 0.05 considered a statistically significant difference.

## 3 RESULTS

### 3.1 YPEL1 Expression is Dysregulated Across Various Cancer Types

We confirmed that YPEL1 was deregulated in most cancers in the TCGA cancer atlas (Fig. **1A**). Specifically, using the GEPIA online tool, we observed that YPEL1 was up-regulated in diffuse large B-cell lymphoma (DLBC) tumor samples (n = 47) compared to normal tissues (n=337) (Fig. **1B**). Further analysis revealed whether YPEL1 was associated with overall survival in cancer patients; high expression of YPEL1 exhibited poor survival while low YPEL1 expression showed better overall survival (Fig. **1C**). Although the difference did not reach statistical significance due to the low sample size, the hazard ratio (HR) for high- and low-expression groups was 1.4 and 0.63, respectively. The HR greater than 1 for the YPEL1 high-expression group indicates a strong association between elevated YPEL1 expression and reduced in patients with NK/TCL.

Immunohistochemical analysis of patient samples from extranodal nasal NK/T cell lymphoma and lymph nodes with reactive hyperplasia revealed upregulation of YPEL1 in NK/T cell lymphoma tissues compared to controls. YPEL1 protein expression showed elevated expression in NK/T cell lymphoma tissues (Fig. **1D**). To further validate these findings, we performed qPCR analysis to measure YPEL1 mRNA expression levels in the samples from both control and NK/T cell lymphoma patients. This analysis corroborated our immunohistochemistry results, showing upregulation of YPEL1 expression in NK / T cell lymphoma patients (Fig. **1E**). Overall, our results indicate that YPEL1 is deregulated in TIME2.0 datasets, including diffuse large B-cell lymphoma (DLBC). High expression of YPEL1 is associated with poor patient survival.

### 3.2 Establishment of YT/Gem-R to Determine the Function of YPEL1

To further investigate the molecular function of YPEL1, we utilized NKTCL cell lines as a model system. Initially, we checked the protein expression of YPEL1 in NK/T cells, SNK-6, NK92, and YT by western blotting and Flow cytometry. The YT cell line exhibited the highest YPEL1 protein expression (Fig. **2A**-**B** and Fig. **S1**). We selected the YT cell le to explore the functions of YPEL1. Given that chemoresistance is prevalent in NKTCL, studying molecular determinants like YPEL1 requires an experimental model of drug-resistant cancer cells. Therefore, we established a YT/Gem-R by continuously exposing the cells to escalating concentrations of gemcitabine. Morphological differences between YT and YT/Gem-R cells were observed, as depicted in Fig. **2C**. We determined the IC50 for YT and YT/Gem-R to be 0.810 μg/ml (R^2^=0.911), and 10.02 μg/ml (R^2^=0.901), respectively (Fig. **2D**). These indicate that YT/Gem-R cells are 13 times more than YT cells. Analysis of YPEL1 protein expression in these cells revealed upregulation of YPEL1 in the YT/Gem-R cells (Fig. **2E**-**F**). YT showed a higher expression of the YPEL1 protein. And YT/Gem-R cell line was successfully established the YT/Gem_R cell line to further investigate the mechanisms underlying drug resistance.

### 3.3 Differential Gene Expression Profiles of YT and gYT/Gem-R

To further investigate the molecular mechanism of YPEL in NK/TCL, we performed RNA sequencing of these two cell lines, YT and YT/Gem-R. Differential expressed genes (DEGs), were shown as a volcano map (Fig. **3A**). Among these DEGs, YPEL1 was notably highlighted. We identified a total of 64 DEGs with a p-value of *P*<0.05 and logFC>2, of which 43 genes were up-regulated and 21 down-regulated (Fig. **3B**). We then performed the Kyoto Encyclopedia of Genes and Genomes (KEGG) pathways and Gene Ontology (GO) enrichment analysis. The GO enrichment analysis is detailed in Figure **3C**. Notably, the top 5 biological processes related to immune responses, such as neutrophil-mediated immunity, were identified (Fig. **3C**). KEGG enrichment analysis corroborated these findings, highlighting significant enrichment in immune-related pathways such as Human T cell leukemia virus 1 infection, Th cell differentiation, cell cycle, cellular senescence, and cell apoptosis (Fig. **3D**). Furthermore, Gene Set Enrichment Analysis (GSEA) revealed significant activation ofIL2/Stat5 signaling, and interferon-gamma response (Fig. **3E**-**G**) with a *P*-value of < 0.05. These data suggest that YT/Gem-R cell lines exhibit significant expression of DEGs associated with immune responses. Fig. **S2**.

### 3.4 YPEL1 Depletion Induces Apoptosis in YT Cells

We investigated the effect of YPEL1 depletion in YT cells *in vitro* by establishing shRNA-mediated YPEL1 knockdown in YT cells (YT-shYPEL1). The efficiency of YPEL1 knockdown in YT-shYPEL1 cells was confirmed through Western blotting (Fig. **4A**) and qPCR (Fig. **4B**). The expression of YPEL1 was depleted using two specially designed shRNAs, and shRNA-2 achieved over 80% knockdown efficiency. The depletion of YPEL1 in YT-shYPEL1 cells led to a significant reduction in cell growth and increased cell death, as demonstrated by the altered cell morphology (Fig. **4C**). Quantitative analysis of cell growth, performed using the CCK-8 assay, revealed more than a 50% reduction in growth (Fig. **4D**). To further explore the effects of YPEL1 depletion, we measured the apoptosis rate using flow cytometry. Approximately 40% of YT-shYPEL1 underwent apoptosis compared to YT cells (Fig. **4E**-**F**). Additionally, cell cycle analysis indicated a growth arrest at G1/S and G2/M phases for YT cells with YPEL1 knockdown (Fig. **4G**-**H**). We also investigated the role of autophagy in YT-shYPEL1 cell, death by staining the cells with the autofluorescent compound monodansylcadaverine (MDC), which labels autophagic vacuoles. No significant difference in MDC labeling was observed between control and YPEL1-depleted cells (Fig. **4I**). These suggest that apoptotic cell death and no involvement of autophagy were associated with YPEL1-depletion.

### 3.5 YPEL1 Depletion Induces Apoptosis in YT / Gem-R

We previously observed that YPEL1 depletion induced apoptosis in YT cells. Here, we investigated whether there were similar findings in YT/Gem-R. We construct shRNA-mediated knockdown of YPEL1 in YT/Gam-R cells. Previously, we observed a higher knockdown efficiency for YPEL1 with shRNA#2; therefore, we used only shRNA#2 for YT/GEM-R cells (YT/GEM-R-shYPEL1) and found similar effects with more than 80% efficiency (Fig. **5A** and **B**). Notably, when YPEL1 depletion in YT/Gem-R cells reduced the IC50 of gemcitabine to 2.42 mg/ml (R^2^=0.950). indicating that the removal of YPEL1 in YT/Gem-R sensitizes these resistant cells to gemcitabine. We also assessed the effects of YPEL1 depletion on the cell activity of YT/Gem-R cells, which showed a significant growth reduction in YT/Gem-R-shYPEL1 (Fig. **5D**-**E**). Additionally, similar to the YT cells, YT/Gem-R-shYPEL1 cells exhibited around 40% apoptosis upon YPEL1 depletion (Fig. **5F**-**G**). Autophagy was examined by MDC staining in YT/Gem-R-shYPEL1 cells revealing a low number of MDC-positive cells (Fig. **5H**). This was also supported by the up-regulation of Atg12 (Fig. **5I**-**J**). We also investigated the impact of YPEL1 depletion on key signaling pathways. Western blot analysis revealed down-regulation of the p38, JNK1/2, ERK1/2, AKT, and NF-kB pathways in both YT and YT/Gem-R cells.. These findings suggest that YPEL1 mediates its effects through these signaling pathways.

### 3.6 *In Vivo* effects of YPEL1 Depletion in the Xenograft Model

To better understand the role of YPEL1, we performed *in vivo* xenograft experiments using nude mice. The tumor growth curve showed that YPEL1 knockdown inhibits the growth of the tumors compared to the sh-control group (Fig. **6A**). Additionally, tumor weight was significantly reduced in the YT-shYPEL1 (Fig. **6B**-**D**). Importantly, no difference was observed in the body weight of mice between the two groups (Fig. **6E**). When analyzing the overall survival of these tumor-bearing mice, we observed that the YPEL1-depleted group showed a better survival rate than the control group (Fig. **6F**). We also confirmed the depletion of YPEL1 in tumors by Western blotting (Fig. **6G**-**H**) and qPCR (Fig. **6I**). In short, our results demonstrate that YPEL1 depletion suppresses tumor growth *in vivo*, although it is associated with poorer survival outcomes in tumor-bearing mice.

## 4 DISCUSSION

Our study shows that YPEL1 is deregulated in various cancers in the TCGA cancer atlas. The correlation between high YPEL1 expression and poor patient survival highlights its potential role as a biomarker for tumor aggressiveness. Conversely, lower expression levels of YPEL1 are associated with improved survival rates, suggesting that YPEL1 may act as an oncogene in certain contexts. Among the various cell lines analyzed, YT cells exhibited higher expression of the YPEL1 protein among different cell lines, and this expression was further augmented in YT/Gem-R. The identification of YPEL1 as a significant factor in chemoresistance is particularly noteworthy. YPEL1 depletion leads to apoptosis in YT and YT/Gem-R underscores its critical role in cell survival mechanisms.. We also found that depletion of YPEL1 suppressed tumor growth *in vivo*.

YPEL1 is a nuclear protein involved in the mesenchymal-epithelial transition during tissue development [9]. The tumor-promoting nature of YPEL1 is supported by its involvement in essential processes such as the mesenchymal-epithelial transition, which is crucial for tissue development and regeneration. The YPEL1 gene was found to be downregulated in pancreatic cancer cells [13] and also associated with reduced proliferation and invasion [16]. The increased expression of YPEL1 in ccRCC, particularly its correlation with tumor purity and infiltration, suggests that YPEL1 may be facilitating the aggressive phenotype of such tumors. YPEL1 is also up-regulated in renal tissues, showing its oncogenic nature [18].

Our investigation on DEGs in YT and YT/Gem-R revealed significant enrichment in immune-related genes. This raises the possibility that YPEL1 may modulate the immune responses in the tumor microenvironment, contributing to the evasion of immune surveillance a critical feature of tumor progression. Future studies should elucidate the precise mechanisms through which YPEL1 modulates immune response, understanding these interactions could pave the way toward novel immunotherapeutic strategies.

Interestingly, our findings indicate that higher expression of YPEL1 correlates with poor survival, recent studies have suggested a paradoxical relationship in ccRCC [18], where decreased YPEL1 expression has been linked to a worse prognosis. This contradiction highlights the complexity of YPEL1's role across different cancer types and emphasizes the need for understanding its function as both a potential tumor promoter and a prognostic marker.

Gemcitabine is an important potent antitumor analog [19]. The establishment of YT/Gem-R provided a robust model for exploring the functional implications of YPEL1. The elevated levels of YPEL1 in YT/Gem-R suggest a possible adaptive mechanism by which tumors develop resistance to therapy. The depletion of YPEL1 expression triggered apoptosis in both sensitive and resistant cells, reinforcing the concept that targeting YPEL1 could enhance the efficacy of gemcitabine and possibly other chemotherapeutics.

Furthermore, the role of autophagy in the context of YPEL1 depletion is intriguing. While we observed significant apoptosis in YT/Gem-R cells, autophagy appears to be selectively activated in these resistant cells. The low number of MDC-positive cells following YPEL1 knockdown suggests that the typical protective role of autophagy in cell survival might be subverted in this context. This prompts further investigation into the balance between apoptosis and autophagy in response to YPEL1 modulation, especially in the backdrop of chemoresistance. We identified key signaling pathways-p38, JNK1/2, ERK1/2, AKT, and NF-κB-as mediators of YPEL1’s effects on cell fate.YPEL1 expression levels were mediated through the p38, JNK1/2, ERK1/2, AKT, and NFκB pathways, as the phosphorylation levels of the indicated proteins decreased in cells depleted with YPEL1. Understanding how YPEL1 interferes with these signaling cascades could reveal new therapeutic targets and enhance our understanding of cancer biology.

*In vivo*, our results showed that YT cells with control shRNA developed significantly larger tumors than YPEL1-depleted cells. The decrease in tumor weight and size in the YPEL1-depleted group further substantiates the notion that YPEL1 is a crucial contributor to tumor growth and progression.

Overall, while our findings establish the role of YPEL1 in promoting tumor growth and survival, further research is necessary to dissect its involvement in invasiveness, migration, and immune infiltration. Exploring these dimensions will deepen the overall understanding of YPEL1’s multifaceted roles in NKTCL, and help in strategizing therapeutics targeting YPEL1 in cancer treatment.

## LIMITATIONS AND CONCLUSION

High expression of YPEL1 was correlated with poor survival, whereas low expression is associated with favorable survival of the patients. Importantly, YPEL1 depletion induces apoptosis in YT cells and YT/Gem-R, indicating its critical role in cell survival mechanisms. Furthermore, our results show that down-regulation of YPEL1 leads to apoptosis and autophagy and that YPEL1 depletion significantly suppresses tumor growth *in vivo*. Given the widespread deregulation of YPEL1 across various cancers, future research should focus on elucidating the potential associations between YPEL1 and other malignancies, which may enhance our understanding of its role in cancer biology and contribute to the development of targeted therapies.

## Figures and Tables

**Fig. (1):**
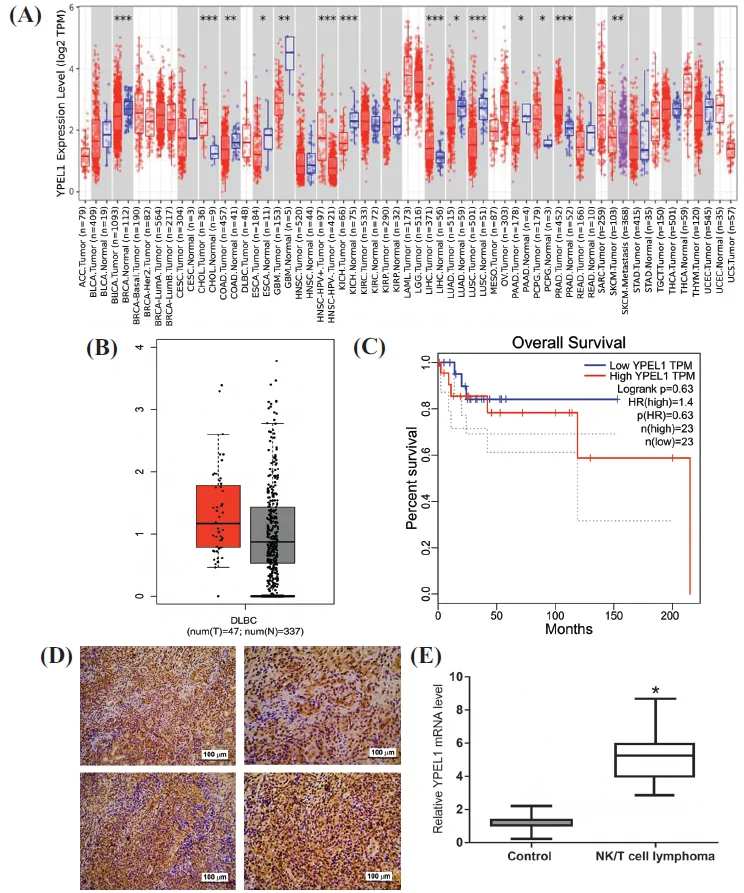
The expression of YPEL1 in different tumors. (**A**) The expression of YPEL1 in different tumors was analyzed by b the TIME2.0 (timer.cistrome.org). (**B**) The expression of YPEL1 in diffuse large B-cell lymphoma was analyzed using GEPIA database (http://gepia.cancer-pku.cn/). (**C**) The relationship between YPEL1 expression and survival rate of patients with NK/T cell lymphoma (http://gepia.cancer-pku.cn/). (**D**) The immunohistochemical analysis of YPEL1 protein expression extranodal nasal NK/T cell lymphoma (upper left and right) and chronic lymphadenitis (bottom left and right). (**E**) The expression of YPEL1 mRNA in NK/T cell lymphoma and chronic lymphadenities. *P<0.05.

**Fig. (2):**
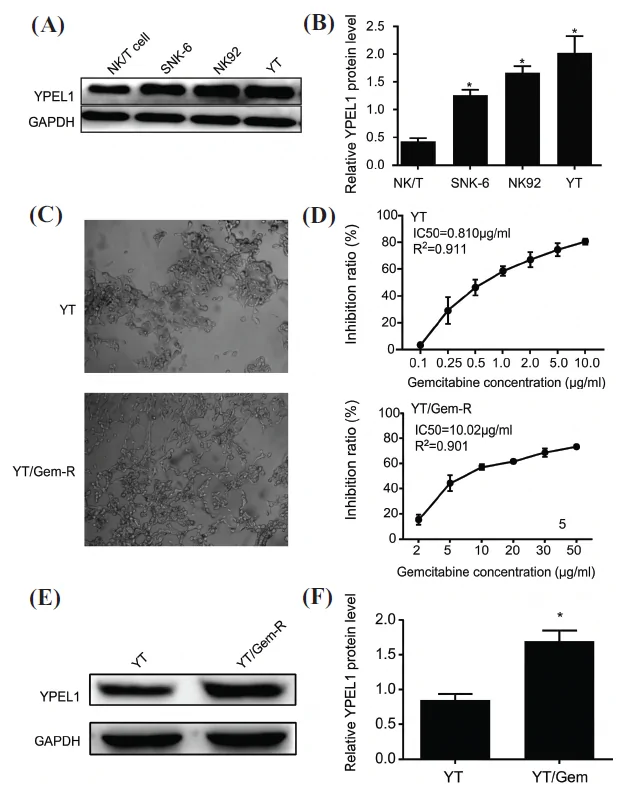
The drug-resistant cells YT/Gem were constructed. (**A**-**B**) The expression of YPEL1 protein in primary NK/T cells and lymphoma cell lines. (**C**) Morphological observation of drug-resistant cells YT/Gem (Image captured at 4× magnification). (**D**) The drug resistance index of YT/Gem cells. (**E**) The expression of YPEL1 protein in YT/Gem cells, detected by Western blot (**F**) Relative quantification was performed using grayscale intensity. *P<0.05.

**Fig. (3):**
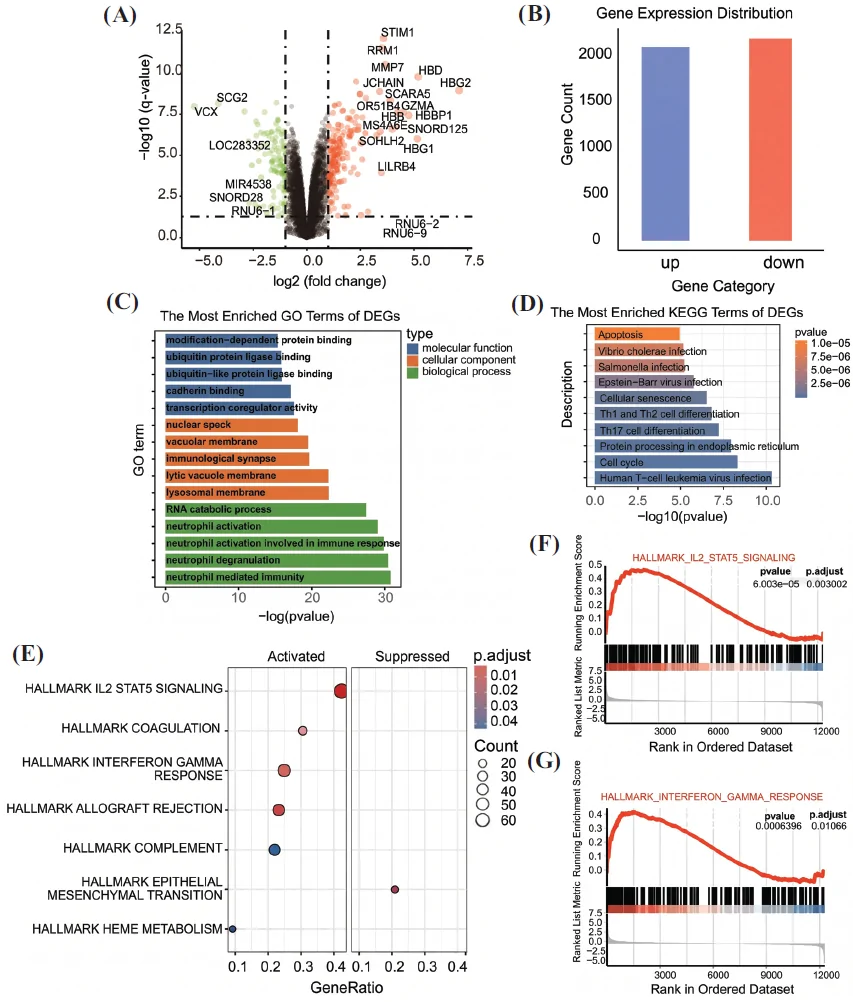
The gene expression differences between YT and YT/Gem according to transcriptome sequencing. (**A**) Volcano map of differentially expressed genes. (**B**) The count of differentially expressed genes. (**C**) GO enrichment analysis of differential genes in YT and YT/Gem-R cells. (**D**) KEGG enrichment analysis of differential genes in YT and YT/Gem-R cells. (**E**) The enrichment analysis of GESA Hallmark signal pathway. (**F**) The IL2-STAT5 signal pathway GSEA analysis. (**G**) The GSEA analysis of IFNγ signaling pathway.

**Fig. (4):**
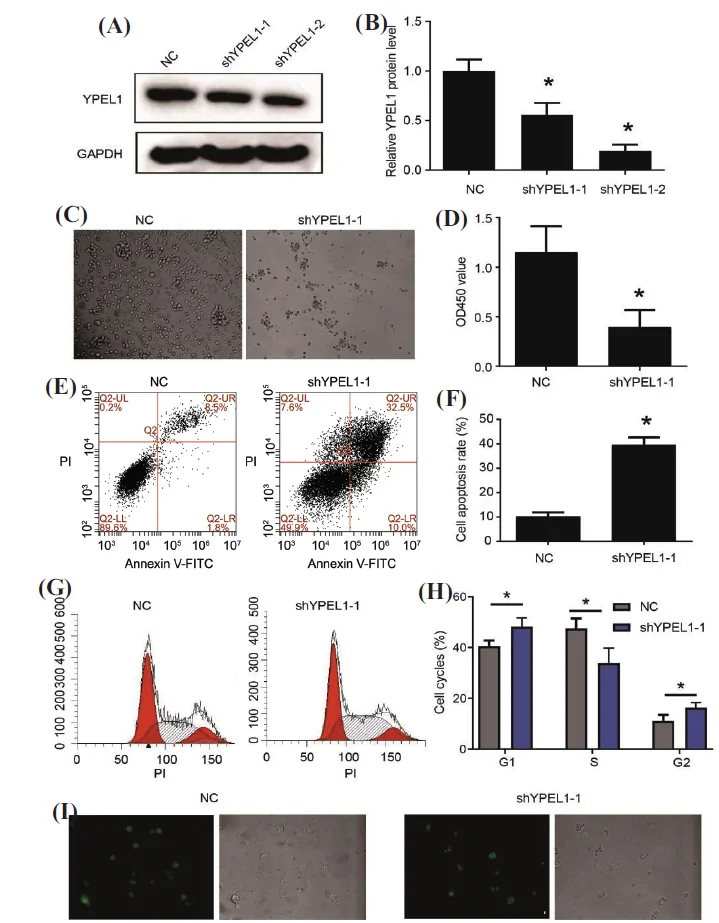
The effect of YPEL1 interference on YT cell activity. (**A** and **B**) The expression of YPEL1 protein in YT cells after infection was determined by western blot. (**C**-**D**). The effect of interfering with YPEL1 on YT cell activity. (**E**-**F**) The effect of interfering with YPEL1 on cell apoptosis. (**G**-**H**) The effect of YPEL1 interference on cell cycle in YT cells. (**I**) The effect of interfering with YPEL1 on YT cell autophagy was determined by MDC. *P<0.05.

**Fig. (5):**
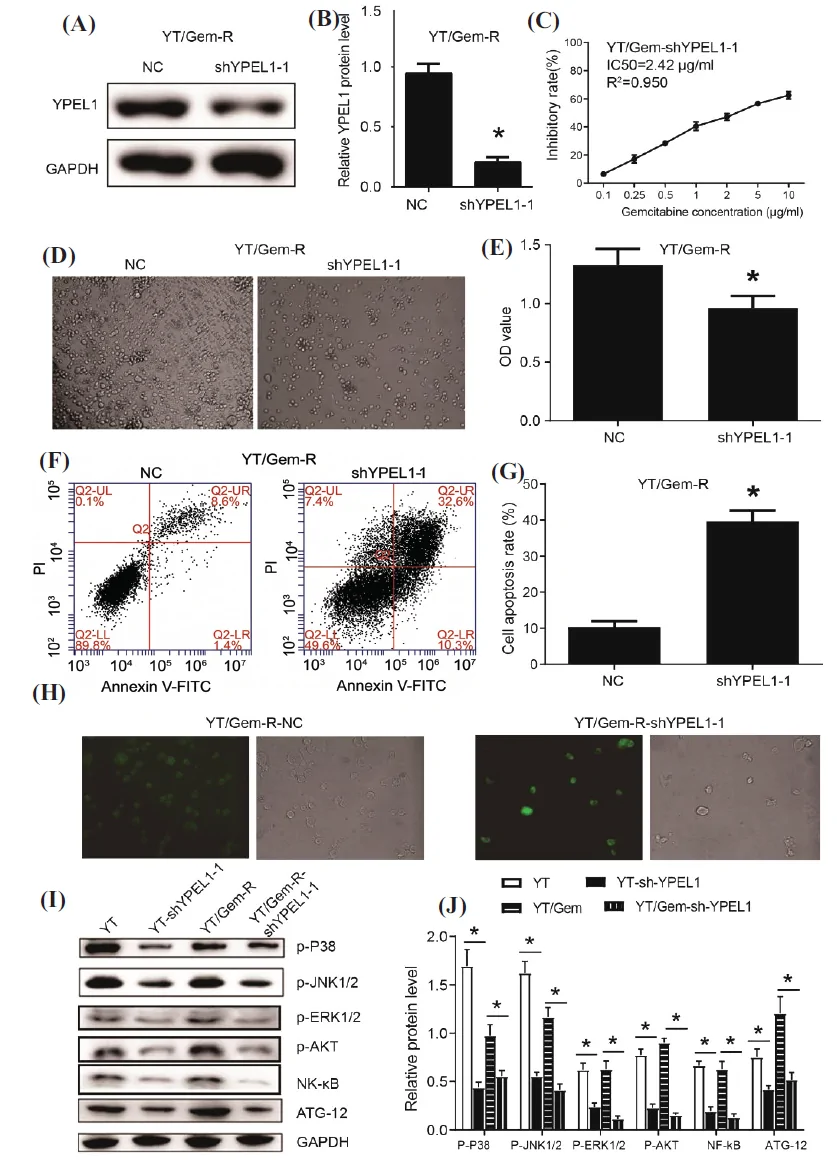
The effect of YPEL1 interference on YT/Gem-R cell activity. (**A** and **B**) The expression of YPEL1 protein in YT/Gem-R cells after YPEL1 knockdown was determined by western blot. (**C**) The influence of YPEL1 interference on drug resistance of YT/Gem-R cells. (**D**-**E**). The effect of interfering with YPEL1 on YT/Gem-R cell activity. (**F**-**G**). The effect of interfering with YPEL1 on cell apoptosis in YT/Gem-R. (**H**) The effect of interfering with YPEL1 on YT/Gem-R cell on autophagy was determined by MDC. (**I**) The expression of different signal pathway-related proteins in each group of cells. *P<0.05.

**Fig. (6):**
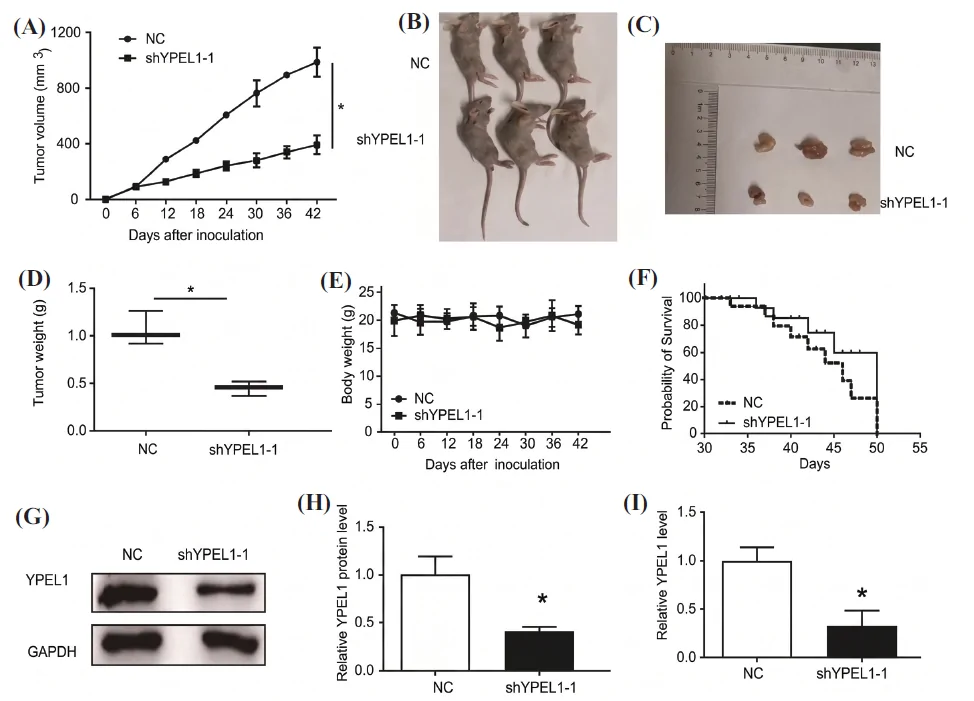
The influence of interfering with YPEL1 *in vivo*. (**A**) The volume statistics of transplanted tumors at different times. (**B**) The photo of transplanted tumors *in vivo*. (**C**) The photo of transplanted tumors from the xenograft model. (**D**) The weight statistics of the transplanted tumor. (**E**) The influence of interfering with YPEL1 on weight changes in tumor-bearing mice. (**F**) The influence of interfering with YPEL1 on survival in tumor-bearing mice. (**G**-**I**) The expression of YPEL1 protein (G) and mRNA (F and I) in tumor tissues. *P<0.05.

## Data Availability

All data generated or analyzed during this study are included in this published article.

## LIST OF ABBREVIATIONS

AKT: Protein Kinase B
Atg12: Autophagy-related Gene 12
CCK8: Cell Counting Kit-8 ccRCC = Clear Cell Renal Cell Carcinoma
DEG: Differentially Expressed Gene
ERK1/2: Extracellular Signal-Regulated Kinase 1/2
GO: Gene Ontology
GSEA: Gene Set Enrichment Analysis
HRP: Horseradish Peroxidase
JNK1/2: c-Jun N-terminal Kinase 1/2
KEGG: Kyoto Encyclopedia of Genes and Genomes
LMO7: LIM Domain Only Protein 7
MDC: Monodansylcadaverine
NF-kB: Nuclear Factor Kappa B
NK/T: Natural Killer/T-cell
NKTCL: Natural Killer/T-cell Lymphoma
OD: Optical Density p38 = Mitogen-Activated Protein Kinase
PBS: Phosphate-Buffered Saline
PI: Propidium Iodide
PVDF: Polyvinylidene Difluoride qPCR = Quantitative Polymerase Chain Reaction
RIPA: Radioimmunoprecipitation Assay buffer
RPMI: Roswell Park Memorial Institute medium
RT-qPCR: Reverse Transcription Quantitative Polymerase Chain Reaction
SDS PAGE: Sodium Dodecyl Sulfate Polyacrylamide Gel Electrophoresis shYPEL1 = Short Hairpin RNA Targeting YPEL1
TCGA: The Cancer Genome Atlas
YPEL1: Yippee Like 1
YT/Gem-R: Gemcitabine-resistant YT Cell Line
