# Supplementary material for: YPEL1 Inhibits Development of Gemcitabine Resistance in NK / T Cell Lymphomas
Source: Curr Cancer Drug Targets. 2025 Mar 17;26(7):702–13. doi: 10.2174/0115680096328745250122231110 (PMC13678611; doi:10.2174/0115680096328745250122231110)
Supplement: Supplementary file 1 [file CCDT-26-7-702_SD1.pdf]

Supplementary Material

YPEL1 Inhibits Development of Gemcitabine Resistance in NK / T Cell Lymphomas

Miao Wang[1,*], Siyu Qian[1,*], Yue Zhang[1], Qingjiang Chen[1], Xudong Zhang[1] and Mingzhi Zhang[1,*]

[1]*Department of Oncology, The First Affiliated Hospital, Zhengzhou University, Zhengzhou, Henan Province, China*

Table S1. Primers list in manuscription.

Primer Names	Sequeces(5'-3')
GAPDH Forward	GGAGCGAGATCCCTCCAAAAT
GAPDH Reverse	GGCTGTTGTCATACTTCTCATGG
YPEL1 Forward	GTCCAAAACTTTCCAAGCGTATC
YPEL1 Reverse	CACGGAATTGAAGAGGTAGGC

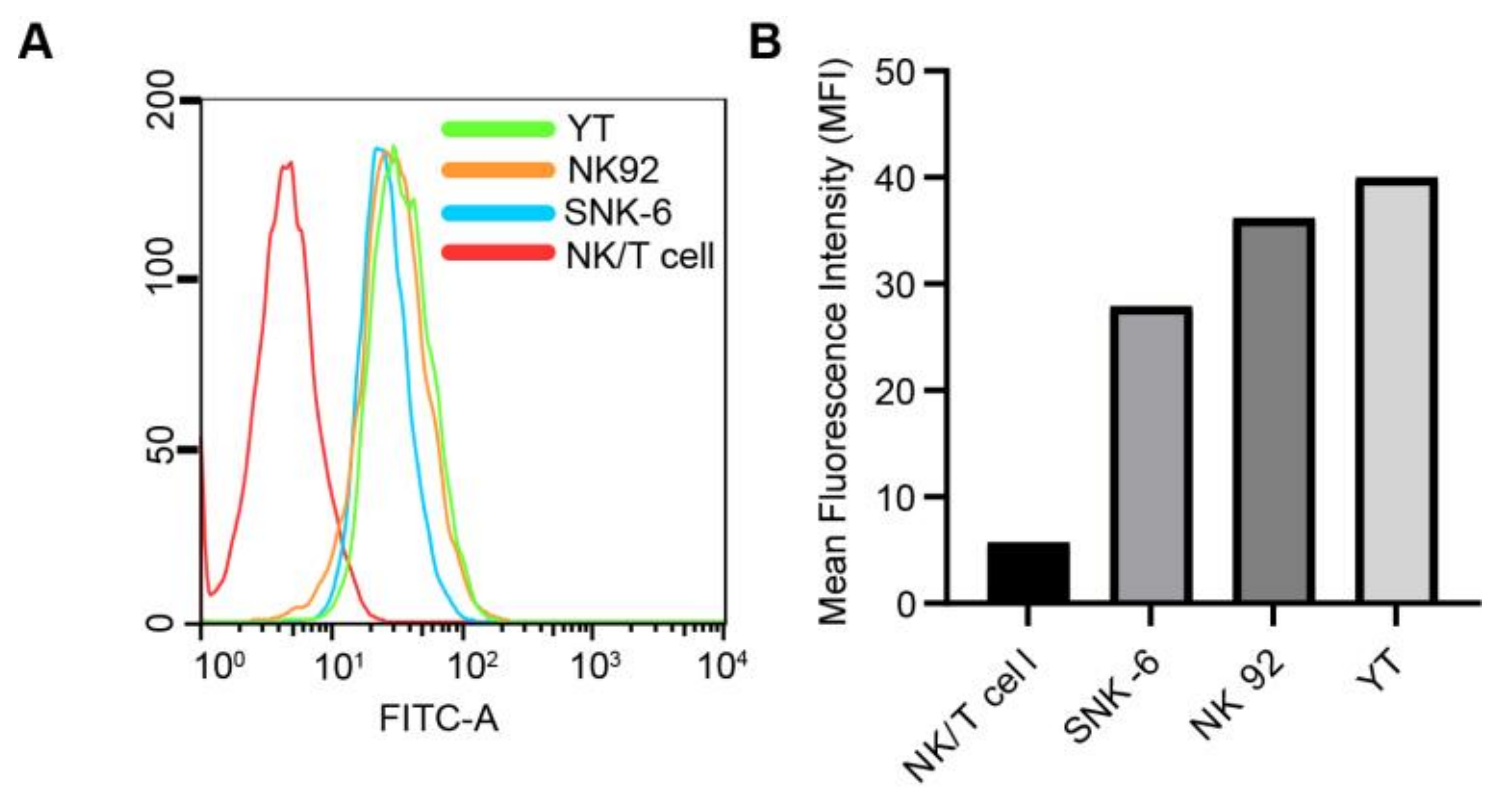

Fig. (S2). The expression of YPEL1 protein in NK/T cell or lymphoma cell lines. A. The expression of YPEL1 protein in NK/T cell and lymphoma cell line determined by cytometry. B. Statistic analysis the expressson quantity of proteins. The cell was fixed with PFA and permeabilized with Triton-X100, stained with the YPEL1 antibody, and then stained with a FITC-conjugated secondary antibody before flow cytometry analysis.
